# Selfitis Behavior: Assessing the Italian Version of the Selfitis Behavior Scale and Its Mediating Role in the Relationship of Dark Traits with Social Media Addiction

**DOI:** 10.3390/ijerph17165738

**Published:** 2020-08-08

**Authors:** Lucia Monacis, Mark D. Griffiths, Pierpaolo Limone, Maria Sinatra, Rocco Servidio

**Affiliations:** 1 Department of Economics, Management and Territory, University of Foggia, 71121 Foggia, Italy; 2 Psychology Department, Nottingham Trent University, Nottingham NG1 4FQ, UK; 3 Department of Humanities, University of Foggia, 71121 Foggia, Italy; pierpaolo.limone@unifg.it; 4 Department of Educational Sciences, Psychology, Communication, University of Bari, 70121 Bari, Italy; maria.sinatra@uniba.it; 5 Department of Cultures, Education and Society, University of Calabria, 87036 Arcavacata di Rende, Italy; rocco.servidio@unical.it

**Keywords:** selfitis behavior, social media addiction, Machiavellianism, narcissism, psychopathy, dark personality traits

## Abstract

Research on selfie-related behavior has recently flourished. The present study expands theoretical and empirical work on phenomenon by assessing the psychometric properties of the Selfitis Behavior Scale among an Italian sample and by examining its unexplored mediating role in the relationships between dark triad traits and social media addiction. A total of 490 participants (53.1% females) completed a self-report survey including socio-demographics, the Selfitis Behavior Scale (SBS), the Short Dark Triad Scale (SD3), and the Bergen Social Media Addiction Scale (BSMAS). Results showed the SBS had a five-factor structure with good psychometrics properties in terms of reliability coefficients and measurement invariance across gender. In addition, findings from the path model supported the mediating role of selfitis behavior in the relationships of narcissism and psychopathy with social media addiction. Machiavellianism was found to be unrelated to selfitis behavior and social media addiction. The model shed light into the previous inconsistent findings on the associations between dark triad traits and social media addiction by taking into account the key role of selfitis behavior as an underlying mechanism. The findings may explain individual differences in personality traits associated with co-dependence (i.e., the combination of the dependence on self and others and social media addiction).

## 1. Introduction

Recently, psychologists’ interest in social media (e.g., Facebook, Menlo Park, California, USA; Twitter, San Francisco, California, USA; Instagram, Menlo Park, CA, USA) has focused on taking and posting digital self-photos (i.e., selfies), and is one of the most popular activities especially among adolescents and emerging adults [1,2,3]. The reason for this growing attention concerns the analysis of what underlies these practices, which powerfully represent the self and impact upon self-esteem in a period relevant to identity formation, self-image, and social interactions [4]. By being taken on digital devices equipped with editing software, selfies can be manipulated by users to how they want to appear and look like, so that they can get feedback in the form of “likes”. Such manipulation could lead to various potential mental health problems and unhealthy online behaviors, such as the onset and maintenance of addictive use of social media.

In 2014, the term “selfitis” was first used in an article in the Adobo Chronicles claiming that American Psychiatric Association (APA) was going to classify a new mental disorder (i.e., “selfitis”), as an obsessive-compulsive desire to take photos and shared them via social media, as a way to make up for the lack of self-esteem and to fill a gap in intimacy [5]. According to the article, there were three levels of the disorder: borderline, acute, and chronic selfitis, depending on how often individuals take selfies and post them on social media. The article turned out to be a hoax, but it sparked research into a practice that is highly popular because “a selfie can say more about a person than the written word” [6] (p. 1), and also facilitated the invention of selfie-sticks.

Selfitis was formally operationalized in 2018 by Balakrishnan and Griffiths [7], who empirically showed the existence of selfitis as a potential behavior to add to technologically-related mental health disorders. They developed a psychometric scale, the Selfitis Behavior Scale (SBS), which classified individuals into one of three categories first outlined in the hoax article (i.e., borderline, acute, and chronic). Balakrishnan and Griffiths’ starting point was the observation that, even though posting selfies allows individuals to express their own self-oriented actions, and establishing their individuality and self-importance, other psycho-social-environmental factors might generate different selfie behaviors. The SBS comprises six sub-components: environmental enhancement (i.e., to feel good and show off to others in specific locations), social competition (i.e., to get more “likes” on social media), attention seeking (i.e., to gain attention from others), mood modification (i.e., to feel better), self-confidence (i.e., to feel more positive about oneself), and subjective conformity (i.e., to fit in with one’s social group and peers). 

Lin et al. [8] provided further empirical evidence of the psychometric properties of the SBS from Persian speaking participants Iran and Afghanistan. The present study follows on from these two previous psychometric studies by (i) assessing the psychometrics properties of the Italian version of the scale, and (ii) testing the mediating role of selfitis in the relationships between dark triad traits and social media addiction. With regard to the first goal, given the lack of a validated measure in the Italian context, the study attempted to ascertain the factorial dimensionality, the reliability, and the external validity of the original scale among Italian adolescents and young adults, as well as its measurement invariance across gender. Consequently, we expected to (i) replicate the six-factor structure of the original version, (ii) show that the estimated factors assessed the same underlying latent construct within each group, and (iii) to establish good internal and external reliability. 

The second goal focused on the relationships between selfie-taking and other predicting constructs that could provide further insight into the underlying mechanisms promoting social media addiction. Among various factors, the personality traits play an important role in the initiation, development, and maintenance of addictive use of social media [9], despite the Big Five conceptualization being limited in explaining this phenomenon [10,11]. Utilizing Uses and Gratification Theory [12], recent studies have tried to account for the different intentions and gratifications as the driving forces in predicting the behavioral intentions of social media users. This theory was also applied to the analysis of individual differences in light of the trait of narcissism aside from the broad Big Five traits. Indeed, social gratifications are rooted in narcissistic impulses that may lead to greater problematic use of social media [13,14]. In this research, narcissism was conceptualized following the Dark Triad Traits framework that includes three socially aversive traits (i.e., narcissism, Machiavellianism, and psychopathy).

As for narcissism, a recent meta-analytic review [15] described three theoretical models predicting elevated social media use by narcissistic individuals. The first is the self-enhancement model, according to which social media represent favorite environments for gaining admiration to fulfill self-enhancement needs and reinforcing the narcissistic self. The second is the fit model, which considers social media use as a means to encourage wide but shallow social networks as a good fit for narcissistic skills and abilities. Individuals high in grandiose narcissism prefer emotionally shallow social relationships and like to associate themselves with high status others [16]. The third is the personality model based on the personality trait construct in terms of the Big Five traits, and posits that individuals, who are characterized by the grandiose quality of narcissism comprising high extraversion and openness, and low agreeableness [17], tend to have more friends and generate more content on social media. 

Although previous research has shown the important role played by narcissistic traits in social media usage [13,18,19], other studies have not reported such [20,21]. A systematic review on this topic carried out by Casale and Banchi [22] reported inconsistent findings ranging from positive to no associations, when investigating problematic social media usage as a unitary category. To overcome this limitation, the authors suggested that specifying different online platforms under the umbrella of social networking sites that might explain differences in people’s motivations for using SNSs. However, a careful inspection of the association between narcissism and problematic social media use across various studies shows that such a relationship becomes significant when considering other potential mediators/moderators. Among them, antisocial online behaviors such as cyberbullying and cyberstalking [23], and self-esteem [24] are the most investigated.

A further mediating mechanism that could promote the addictive use of social media is selfie-related behaviors, which have been recently associated with narcissism [25,26,27,28,29,30,31]. Individuals with elevated narcissistic traits manage and promote their online self throughout the day by taking and sharing selfie content utilizing the gratifying medium of social media in order to gain others’ attention and admiration for preserving their own fragile self-image. This behavior could be a possible cause for their frequent and intense use of social media, and the escalation into addiction for a small minority of individuals [32,33]. Although significant mediating effects of selfie-related behaviors between narcissism and problematic smartphone use have been reported [34], it should be noted that the measure of selfie-related behaviors was ad hoc and created without any standard psychometric assessments. 

In light of the aforementioned theoretical models and empirical evidence, the present study hypothesizes there will be positive associations of narcissism with selfitis behaviors (H1) and social media addictions (H2), and the mediating role of selfitis behaviors in the associations between the trait and social media addiction (H3). Beyond narcissism, the other two dark traits, Machiavellianism and psychopathy considered on a subclinical level, were taken into account in association with selfitis behavior and social media addiction. According to the Dark Triad model, the “darkness” is captured by shared use of antagonistic, dishonest, and egocentric interpersonal styles, which reflect disagreeableness according to the Five Factor Model. Being socially aversive personality traits, Machiavellianism and psychopathy (like narcissism) may be characterized by reward and sensation-seeking [35]. Individuals scoring high on these traits tend to manipulate their physical appearance by posting and sharing their own selfies to achieve social gains [36,37], and are more likely to be addicted to social media.

As for psychopaths, they are often impulsive, reckless, and more willing to engage in antisocial online behaviors [38], as well as using social media platforms, where they can express their hostile and aversive attitudes without fear of punishment [39]. As for Machiavellians, they tend to demonstrate strategic planning in social interactions, employing more self-presentation tactics on social media platforms [40]. To our knowledge, there are only a few studies examining the associations between the three dark traits with social media addiction and they have shown contrasting findings. While Machiavellianism has generally been found to be unrelated to social media addiction [41], some associations have been found with problematic internet use [42,43]. Psychopathy has consistently been associated with problematic social media and internet use [11,43,44,45]. By contrast, and as already highlighted, there have been both significant and non-significant associations found between grandiose narcissism and social media addiction [22,41].

In light of the aforementioned conflicting results, and given that there is still a dearth of research examining a conceptual framework for the relationship between dark triad traits, selfitis behavior, and social media addiction, the second goal in the present study was to test a mediational model. It was hypothesized that, like narcissism, psychopathy would be positively associated with social media addiction (H4) and selfitis behavior (H5), and that the association between psychopathy and social media addiction would be mediated by selfitis behavior (H6). In addition, it was hypothesized that there would be no association of Machiavellianism with social media addiction (H7) and selfitis behavior (H8), and, consequently, no mediating role of selfitis behavior (H9). In agreement with the Goals-Planning Action Model (GPA) [46,47] which is focused on the interpersonal influence on the basis of different secondary goals, individuals scoring high on Machiavellianism (i.e., being strategic, skilled manipulators, and dealing with others in an instrumental way), are more concerned with self-oriented secondary goals (e.g., personal resource and arousal management goals) and may prefer face-to-face channels of communication to manipulate others and to get immediate rewards.

Finally, examining the mediating associations between Dark Triad traits and social media addiction requires the inclusion of gender as an important variable to get the full picture, since significant gender effects were generally found among the aforementioned associations. Some studies have reported that girls usually share and post more selfies on social networks than boys [30,34,48,49,50], and other studies have reported an association between narcissism and selfie-posting and editing behavior among men [27,31,48]. Similarly, Arpaci et al.’s study [48] demonstrated the influence of selfie behavior on narcissism but only in men. Conversely, no differential role of gender was reported in the relationship between narcissism and online photos [51]. Given the inconsistent findings related to gender, the present study also examined this variable in relation to the aforementioned relationships.

## 2. Materials and Methods

### 2.1. Participants, Procedure, and Ethics

A convenience sample comprising 490 participants (M_age_ = 21.23 years, SD = 3.57; 53.1% females) was recruited from the highest year of secondary schools and from different degree university courses (from humanistic to scientific courses). Participants completed a paper-and-pencil self-report survey, which took approximately 15 min. 

The schools and university were chosen on the basis of their availability to participate (i.e., convenience sampling), and students were randomly selected from a pool of classes. The procedure was shared among researchers, headmasters, and teachers. A trained researcher was present during the sessions to explain the procedure. During the data collection in the schools, teachers were present in classrooms, but they did not talk to students. As for university participants, they were recruited in the campus (e.g., at the lessons with the support of colleagues, during lecture breaks, at the library, etc.).

After informing participants that taking part in the study was voluntary and anonymous, they gave informed consent according to the Helsinki declaration and the ethical rules of the Italian Psychological Association. Approval for the study was granted by the research team’s university ethics committee. No formative credits or other remunerative rewards were given for participation. Data collection took place during October to December 2019. The Selfitis Behavior Scale was translated from English into Italian separately by the authors of the present study. The translated versions were compared and back-translated into a single English version by a native speaker following the standard guidelines from Beaton et al. [52]. The two versions showed no discrepancies.

### 2.2. Measures

Socio-demographics: The survey included questions concerning gender, age, cigarettes usage (whether the participant smoked more than three cigarettes per day), alcohol use (whether the participant had ever consumed alcohol), soft drug consumption (whether the participant had ever used soft drugs), number of selfie posts per day, and number of friends on social networks.

### 2.3. Selfitis Behavior

The Italian translation of the scale (SBS) [7] was used to assess selfitis behavior (see Appendix A). The SBS comprises 20 items. Each item is rated on a five-point Likert scale ranging from 1 (strongly agree) to 5 (strongly disagree) and includes six subdomains: environment enhancement (e.g., “I take selfies as trophies for future memories”), social competition (e.g., “Taking different selfie poses helps increase my social status”), attention seeking (e.g., “By posting selfies, I expect my friends to appraise me”), mood modification (e.g., “Taking selfies instantly modifies my mood”), self-confidence (e.g., “I feel confident when I take a selfies”), and subjective conformity (e.g., “When I don’t take selfies, I feel detached from my peer group”). A higher score indicates higher levels of selfitis behavior. 

### 2.4. Short Dark Triad (SD3)

The 27-item Italian version of the SD3 [53] was used to assess three socially aversive personality traits: narcissism (nine items, e.g., “People see me as a natural leader”), psychopathy (nine items, e.g., “People often say I’m out of control”), and Machiavellianism (nine items, e.g., “Most people can be manipulated”). The instrument is a non-clinical measure of the Dark Triad and evaluates empirical associations in normal populations. Each item is assessed on a five-point ordinal scale, ranging from 1 (strongly disagree) to 5 (strongly agree). Three mean scores are calculated. Internal consistency reliability estimates for the present study were 0.71 for narcissism, 0.67 for Machiavellianism, and 0.68 for psychopathy.

### 2.5. Bergen Social Media Addiction Scale (BSMAS)

The Italian version of the scale (BSMAS) [54] was used to assess social media addiction over the past year. The instrument contains six items (e.g., “How often during the last year have you tried to cut down on the use of social media without success?”) reflecting core addiction elements (i.e., salience, mood modification, tolerance, withdrawal, conflict, and relapse) [55]. Each item is rated on a five-point Likert scale from 1 (very rarely) to 5 (very often). A total score is calculated with higher scores indicating greater social media addiction. The scale showed good internal consistency in the present study (α = 0.76).

### 2.6. Statistical Analyses 

Descriptive statistics were performed to analyze participants’ socio-demographic variables. The skewness and kurtosis analyses of all items provided indication of non-normality, therefore robust methods were used. First, the factor structure of the Selfitis Behavior Scale was evaluated using confirmatory factor analysis (CFA). CFA was performed with the mean and variance adjusted maximum likelihood (MLM) estimation method, which has been demonstrated as robust with non-normal data. Multiple indices were used to evaluate model fit (adopted cut-offs in parentheses): the chi-square (χ^2^) test value with the associated *p*-value (*p* > 0.05), comparative fit index (CFI ≥ 0.95), Tucker–Lewis Index (TLI ≥ 0.95), root-mean-squared error of approximation (RMSEA ≤ 0.06) and its 90% confidence interval, and standardized root mean square residual (SRMR < 0.08). Second, a parallel factor analysis was carried out to investigate the factor structure of the SBS, using ‘principal axis factoring’ as the extraction method with “oblimin” rotation. Preliminary the Kaiser–Meyer–Olkin (KMO) measure of sampling adequacy and Bartlett test of sphericity were performed to examine the factorability of the data. 

Third, CFA analyses were performed to confirm the emerging factor solution and a second-order solution. The two models were then compared with the following information theoretic indices—Akaike Information Criterion (AIC) and Bayesian Information Criterion (BIC)—with lower values indicating a more parsimonious model. Fourth, Multigroup Confirmatory Factor Analysis (MG-CFA) [56] was performed to test its measurement invariance across gender groups on a set of nested models. For this purpose, a baseline model for each group was determined taking into account the robust statistics (MLM, χ^2^ S-B) and models were statistically compared using the difference in the chi-square-statistics and degrees of freedom. Invariance was tested for configural, metric, and scalar. The ΔCFI value was used to test the between-group invariance of CFA models. According to Cheung and Rensvold [57], the invariance can be assumed when this value is 0.01 or less, in absolute values. As the classical approach based on the χ^2^ difference is sensitive to model complexity and sample size, comparing two nested models is recommended using cut-off values of ΔCFI  < 0 .01 and ΔRMSEA  <  0.015 for metric and scalar invariances [57,58], or other alternative fit indices (e.g., RMSEA, SRMR; [58,59]. Consequently, two models are considered to provide equivalent fit when the following criteria are satisfied: ΔCFI ≤ 0.010, ΔRMSEA ≤ 0.015, and ΔSRMR ≤ 0.010, even when the chi-square difference test is significant. Modification indices (MIs) were analyzed to detect the source of non-invariance when full metric and/or scalar invariance was not established [60].

Fifth, internal consistency was computed using Cronbach’s alpha (α) and McDonald’s (ω) on the dimensions extracted by the parallel analysis. Sixth, construct reliability was examined by assessing convergent and discriminant validity. The former validity included values of Average Variance Extracted (AVE), i.e., the extent to which the items of a specific factor converge or share a high proportion of variance [61], and Composite Reliability (CR), i.e., the shared variance among a set of observed variables that measure an underlying construct. Values of AVE greater than 0.50 and values of CR above 0.60 are considered adequate. The latter validity used the square root of AVE compared with the squared inter-factor correlation among factors. Level of square root of AVE should be greater than the correlations involving the constructs [62]. Another parameter to evaluate construct reliability was the Factor Determinacy (FD) coefficient that refers to the correlation between the estimated and true factor scores. This ranges from 0 to 1 and describes how well the factor is measured, with 1 being the best value [63]. The larger the coefficients (e.g., ≥0.70), the more stable the factors, whereas low coefficients mean that the factors are poorly defined by the observed variables [64]. Seventh, correlational analyses were carried out to assess the concurrent validity of the SBS with a theoretically related construct (social media addiction) utilizing the Bergen Social Media Addiction Scale [BSMAS]. Since taking and sharing photos are frequently carried out in social media, selfitis may be associated with social media addiction [2]; the criterion-related validity of SBS with two criteria comprising the number of posted selfies (per day) and number of friends on social networks (Facebook, Twitter and Instagram). Both validities were performed with bootstrapped correlations with 95% bias-corrected and accelerated (BCa) confidence intervals.

Eighth, a t-test was run to examine gender effects among the variables of interest. Ninth, bivariate and partial correlations were performed among the variables to explore possible mediating effects of selfitis behaviors between dark triad personality traits and social media addiction. Following Furham et al.’s suggestions [65], partial correlations were run to factor out the influence of the other two traits in the patterns of associations between SBS and BSMAS. By doing this, the independent contribution of each trait to the outcome variables was found. Based on the observed associations, a structural equation model (SEM) approach was applied to test the hypothesized mediating role of selfitis behavior. To mitigate possible effects of non-normality of the data and to satisfy the principle of parsimony, the item parceling procedure was undertaken for the sub-scales of the dark personality traits using the item-total correlations assignment method [66]. Gender was also included as a covariate, since previous studies had reported significant effects. 

## 3. Results

### 3.1. Preliminary Statistics

Descriptive statistics of healthy lifestyles showed that 75.1% of participants were non-smokers, 26.2% non-drinkers, and 91.1% did not use soft drugs. Data from online behaviors indicated that 51.3% took and posted one selfie per day, 9.7% took five selfies per day, and 3.4% took more than five selfies per day. Most of the participants declared to have more friends/followers on Instagram (75.5%) and Facebook (67.3%) than on Twitter (6.1%). The mean, standard deviation, skewness and kurtosis were calculated for each item of the SBS. Most of the asymmetry and kurtosis values were below, or slightly above, |1.50| [67], except for the Item 18, which showed slightly high asymmetry (1.78) and kurtosis values (2.82). In addition, the index of multivariate kurtosis K of Mardia [68] was equal to 80.26 (*p* < 0.001). This value indicated a violation of multivariate normality, suggesting the use of robust parameter estimation methods.

### 3.2. Confirmatory, Parallel and Invariance Analysis

First, a confirmatory factor analysis was run to replicate the original factorial dimensionality. Since results were not adequate, χ^2^(S-B) = 431.42, df = 155, *p* < 0.001, CFI = 0.912, TLI = 0.892, RMSEA 90% (CI) = 0.062 (0.05, 0.07), SRMR = 0.048, a parallel factor analysis was performed to a better trimming of its structure and to identify the numbers of factors and their item loadings by using ‘principal axis factoring’ as extraction method with “oblimin” rotation. The Kaiser–Meyer–Olkin (KMO) measure of sampling adequacy was 0.95, indicating that the current data were suitable for the analysis. Similarly, Bartlett’s Test of Sphericity was significant (*p* < 0.001), demonstrating enough correlation between the variables to proceed with the analysis. The results of the parallel analysis indicated the presence of five main factors (Table 1), which fitted well to the data: χ^2^ = 223.62, df = 100, *p* < 0.001, TLI = 0.952, RMSEA 90% (CI) = 0.051 (0.04, 0.06). 

However, the obtained factor structure was different from that of the original study. The first factor explained 12.12% of the variance, the second 13.89%, the third 12.09%, the fourth 10.79%, and the fifth 7.45%. The five factors cumulatively accounted for 56.35% of the total variance, exceeding the 50% recommended for a meaningful factor solution [69]. Confirmatory factor analysis was carried out to test the factorial structure obtained from the parallel analysis. Since data violated the normality condition, maximum likelihood estimation (MLM) was used with robust standard errors to estimate parameters with a correction of chi-square and standard errors [70]. The tested model provided a good fit to the data: χ^2^(S-B) = 344.37, df = 160, *p* = 0.000, CFI = 0.950, TLI = 0.940, RMSEA 90% (CI) = 0.050 (.04, 0.05), SRMR = 0.043. All factor loadings were significant and ranged from 0.60 to 0.91 (Table 1).

A second-order structure indicated fit values equal to the five-factor solution: χ^2^(S-B) = 351.59, df = 165, *p* = 0.000, CFI = 0.949, TLI = 0.942, RMSEA 90% (CI) = 0.049 (0.04, 0.05), SRMR = 0.044. Values of AIC equal to 22909.525 and BIC equal to 23116.735 for the five-factor solution, and AIC equal to 22910.244 and BIC equal to 23096.733 for the second-order solution indicated that both factor structures were equally parsimonious and interchangeable. Finally, findings from MGCFA across gender showed that the equivalence was supported at all levels (i.e., configural, metric and scalar). For the last level, although the Δχ^2^ value was significant, the other three parameters showed values falling within the aforementioned range of recommendation (Table 2).

### 3.3. Scale Reliability

Table 1 shows reliability coefficients—Cronbach alphas (α) and McDonald’s omega (ω)—for the internal consistency reliability, the average variance extracted (AVE) and composite reliability (CR) for the convergent validity, and squared root of AVE for the discriminant validity. Data showed generally good values of the internal consistency (αs and ωs) in spite of the lower value for the fifth factor comprising just two items. As for the convergent validity, findings were generally satisfactory, even if the first factor yielded an AVE value below 0.50 and a CR value higher than 0.70, and the fifth factor showed a lower value of AVE and a CR close to 0.60. Following Fornell and Larcker’s suggestions [62] on AVE less than 0.5 and CR higher than 0.6, it was assumed that the convergent validity of the construct was still adequate. Results from discriminant validity showed good values. Finally, the average of the factor score determinacy coefficients was 0.92, showing an excellent degree of internal consistency. 

### 3.4. Concurrent and Criterion-Related Validity

Results from bivariate correlations showed positive associations between SBS total score and BSMAS, as well as positive associations between each factor of the scale and BSMAS (>0.30, *p* < 0.001; average of correlation was 0.42) (Table 3). Similarly, results from the criterion validity related to the associations of the SBS overall score and its five dimensions with the number of selfie posts and of friends on social media generally indicated positive associations (Table 3 and Table 4).

### 3.5. Gender Differences

To examine gender differences among the variables of interest, t-tests were performed. Findings showed significant gender effects on the three dark traits scores, i.e., Machiavellianism, t(488), 4.416, *p* < 0.001, narcissism, t(488), 4.441, *p* < 0.001, and psychopathy, t(488), 4.679, *p* < 0.001, on the total SBS score, t(488), −3.113, *p* < 0.001, and on BSMAS score, t(488), −3.194, *p* < 0.001. Compared to females, males obtained higher mean scores on Machiavellianism (M = 3.15 SD = 0.630; M = 2.90 SD = 0.658), and narcissism (M =3.07 SD = 0.471; M =2.88 SD = 0.477) and psychopathy (M = 2.54 SD = 0.612; M = 2.27 SD = 0.646), whereas females obtained higher mean scores on SBS score (M = 10.54 SD = 3.65; M = 9.53 SD = 3.48) and BSMAS score (M = 2.19 SD = 0.82; M = 1.96 SD = 0.71).

### 3.6. Zero-Order and Partial Correlations

Correlational analyses were carried out for the total sample and then separately for each group to examine the patterns of associations among the variables of interest. Different associations emerged when comparing zero-order associations in the entire sample and gender groups. In the total sample and female group, both SBS and BSMAS were positively associated with the three dark traits. In the male group they were unrelated with Machiavellianism and positively related with psychopathy. The patterns of association with narcissism changed among males. Narcissism was positively associated with SBS and unrelated with BSMAS. Mixed results emerged when examining the partial correlations for the total and for each gender group. When considering narcissism by controlling for the effects of the other two dark traits, its positive associations with SBS were confirmed among the total sample and among both gender groups, whereas its positive association with BSMAS was found only among females. For Machiavellianism, data confirmed this trait was unrelated to SBS and BSMAS in the total sample and in the gender group. As for psychopathy, its positive associations with SBS and BSMAS were confirmed in the total sample and only in the female group, whereas its relationship was found to be positive with SBS and non-significant with BSMAS among males. Therefore, these relationships were similar to those of narcissism (Table 5).

### 3.7. Mediation Analysis

**Figure 1:**
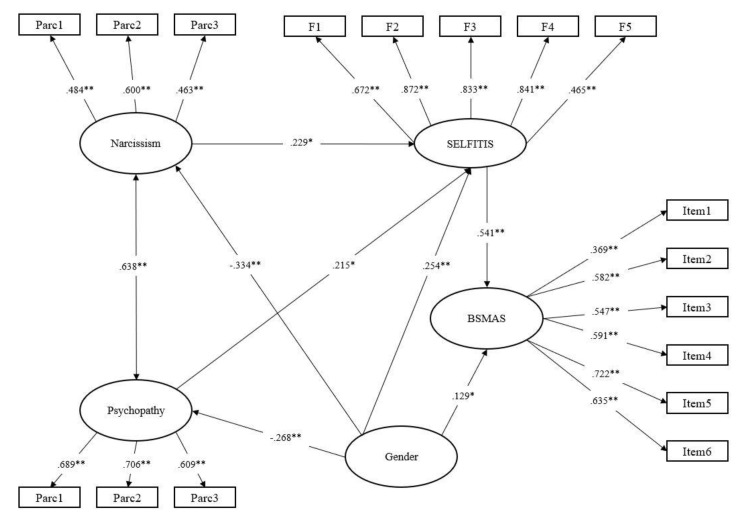
Selfitis as mediator in the relationships between narcissism, psychopathy, and social media addiction. *Note.* Full standardized coefficients are reported; *p < 0.05. **p < 0.001.

A path model was tested using latent variables based on the structural equation model (SEM) approach to examine the mediating role of selfitis behavior between dark traits and social media addiction. Gender was also included as a control variable in the model. Machiavellianism was removed from the analyses being that it was unrelated to SBS and BSMAS. The results of the model showed an adequate fit to the data, χ^2^(S-B) = 235.31, df = 124, *p* < 0.001, CFI = 0.953, TLI = 0.942, RMSEA 90% (CI) = 0.043 (.03, 0.05), SRMR = 0.05. The model explained 54% of the variance in BSMAS and SBS. As shown in Figure 1, all the standardized regression coefficients among the variables were statistically significant. Narcissism and psychopathy showed only significant indirect effects on BSMAS via SBS, β = 0.124, SE = 0.062, *p* < 0.05 and β = 0.116, SE = 0.058, *p* < 0.05, respectively, being the direct paths not significant. In summary, SBS fully mediated the association between narcissism, psychopathy, and BSMAS.

## 4. Discussion

The aim of the present study was twofold: (i) to empirically test the psychometric properties of the SBS among an Italian sample, and (ii) to explore the mediating role of the selfitis behavior in the relationships between dark triad traits and social media addiction. In light of the lack of studies focused on selfitis behavior within Western countries, the first goal of the present study was to address this empirical gap by assessing the psychometric validity and the factor structure of the SBS in an Italian sample. Contrary to expectations, the original six-factor structure was not supported, although the total number of the items of the scale remained equal. Analysis provided evidence for both first- and second-order structures with five-factor solution. The factor loadings were statistically significant, demonstrating that indicators were adequate reflections of their constructs. When looking at the two versions of the scale, many differences emerged in the distribution of the items, therefore new labels for each dimension on the basis of the semantic analysis of item content is now provided.

The first factor was labelled as social and emotional subjective wellbeing because it comprises items reflecting the individuals’ evaluation of a series of emotional states experienced by themselves (e.g., “Taking selfies gives me a good feeling to better enjoy my environment” or “I am able to reduce my stress level by taking selfies”) and within social bonds (e.g., “I gain enormous attention by sharing my selfies on social media” or “Sharing my selfies creates healthy competition with my friends and colleagues”). Following the social interactional theory of emotions [71], selfie-taking and posting behavior imply social interactions, through which individuals can maintain, obtain, or lose specific benefits or rewards, thus experiencing emotional states. The second factor was labelled as self-confidence, being related to self-reinforcement function of selfies [3] that increases individuals’ self-confidence (e.g., “I feel confident when I take a selfie” or “I feel more popular when I post my selfies on social media”). The third factor, self-presentation, encapsulates items referring to the need to belonging and being part of a group (e.g., “If I don’t take selfies, I feel detached from my peer group” or ” I gain more acceptance among my peer group when I take selfie and share it on social media”) in order to increase one’s own social status (e.g., “Taking different selfie poses helps increase my social status”) and to become more attractive and desirable than others (e.g., “I use photo editing tools to enhance my selfie to look better than others”). The fourth factor, need for reassurance/self-approval, includes items showing the individuals’ tendency to use selfies as a mean to validate their own existence thanks to the positive feedback obtained by others (e.g., “I post frequent selfies to get more ‘likes’ and comments on social media” or “By posting selfies, I expect my friends to appraise me”) and by themselves (e.g., “Taking selfies instantly modifies my mood” or “I take more selfies and look at them privately to increase my confidence”). This factor could be related to statement “Videor, ergo sum”, that synthetizes the need to gain the attention from others, to look at and evaluate oneself on the basis of one’s own appearance. The fifth factor, autobiographical memories/documentation, reflects items related to autobiographical memories, and suggests that selfies can be used as social or personal souvenirs of particular experiences (e.g., “Taking selfies provides better memories about the occasion and the experience” or “I take selfies as trophies for future memories”).

The revised factor structure of the Italian version of the SBS was partially in line with the three-factor solution of the Selfie Motivations Scale (SMS) developed by Etgar et al. [3]. Indeed, the Italian version comprises items reflecting semantically the main motivators underlying the self-reinforcement effects, i.e., self-approval, belonging and documentation. In addition, a closer inspection of the Italian dimension self-presentation, could correspond to belonging dimension of the SMS, indicating that items further accounted for the aspects of the conformity and social norms that were lacking in the Israeli version, as affirmed by the aforementioned authors. Likewise, the dimension self-confidence emerging in the Italian version was also related to the self-reinforcement effects of selfies, given that the private and public selfie activity (i.e., taking and posting selfies) has positive effects on self, that is, higher levels of self-confidence. Finally, the dimension social and emotional subjective wellbeing implies that selfie-taking and posting selfies, and being socially-orientated, elicits emotional states experienced by individuals.

In terms of measurement invariance, findings provided evidence of configural, metric, and scalar invariances for gender groups, indicating that the items and the underlying constructs had the same meaning among males and females. Concerning the reliability of the scale, results generally supported its internal consistency as assessed by several indicators, whose values were found to be high. As for construct validity, if AVE and CR values showed adequate convergent validity despite critical values of the fifth factor probably due to the limited number of items, values of square root of AVE supported discriminant validity completely because they were larger than the inter-construct correlations. In other words, the individual constructs were discriminated from each other. In line with other studies [7,8], results demonstrated that the scale could be considered reliable and accurate, although further studies should not only better scrutinize the items reflecting the dimension of documentation/autobiographical memories, but also replicate these findings in cross-cultural contexts to confirm the construct validity.

In terms of external validity, data confirmed the concurrent validity of the instrument showing moderate positive associations of the SBS with its theoretically related construct (i.e., social media addiction). In contrast to Lin et al.’s study [8] reporting mostly weak correlations below 0.30, the expected associations were in line with the idea that selfitis behavior is directly connected to social media, since individuals can fulfil their social needs, such as self-presentation and the need to belong to. The observed positive associations were also in line with studies demonstrating that selfie-sharing on social media improves one’s self-esteem/mood through “likes” [72] and is related to self-presentation behaviors and relationship construction [30,73]. Similarly, and consistent with the authors’ expectations, the positive associations between SBS and the number of daily selfies and of friends/followers on social media provided evidence for the criterion-related validity, although correlation values were weak probably due to the lack of any accurate psychometrics measures of the two criteria. Nevertheless, the positive associations validated the general assumption that a profile owner is perceived to be more socially attractive when he/she has more social media friends [74].

The second goal of the present study aimed at exploring the mediating role of selfitis behavior in the relationships between dark triad personality traits and social media. Overall, the hypotheses were confirmed. First, the positive narcissism-selfitis behavior association (H1) and psychopathy- selfitis behavior association (H4) were confirmed by zero-order and partial correlations in the total sample, males, and females, whereas the positive narcissism-social media addiction association (H2) and psychopathy-social media addiction association (H5) were partially supported. Findings from partial correlations confirmed H2 only in females and H5 in the total sample and females. Therefore, the associations of both traits with social media addiction among males were not significant. These first results could suggest that selfitis behavior is a popular activity among individuals with high scores on both traits irrespective of gender effects, whereas the tendency to become addicted to social media was strictly related to being female with high scores on these traits. 

Second, as for Machiavellianism, when holding constant the other two traits, findings supported the hypotheses, given the lack of its association with selfitis behavior (H7) and social media addiction (H8) in the total sample, male and female groups. Conversely, when considering the zero-order correlations, the positive associations of Machiavellianism with SBS and BSMAS emerged among the total sample and in the female group. Results could shed light into the inconsistent findings between this trait and social media addiction that were previously reported. In contrast with studies showing positive associations [40,45], data corroborated these studies finding no significant association [27,44]. Indeed, because Machiavellians show an ego-drive (i.e., the tendency to derive satisfaction from successful persuasive attempts, and an independence tendency), for them, selfitis activities are not likely to be employed as tactics and strategies on social media, probably given their strong sense of independence. Consequently, the lack of a co-dependence (the combination of both dependence/addiction) was empirically supported by non-significant associations of Machiavellianism with SBS and BSMAS in partial correlations among all three groups (H9).

The hypothesized mediating role of selfitis behavior in the relationships between narcissism and social media addiction (H3) and between psychopathy and social media addiction (H6) was confirmed. Individuals scoring high on narcissism and psychopathy, and being more focused on their individual self-concept, are motivated by interaction-oriented secondary goals (that is, identity and interaction goals, both reflecting the desire to conform to a certain standard of behavior, as well as relational resource goals focused on relationship management) and show much concern for others. 

Both narcissists and psychopaths are ego-driven, but unlike Machiavellians, they are self-vs-others-oriented, since they tend to satisfy their self-reinforcement by dealing with others in an emotional way, and therefore depend on others. Similarly, the primary dependence is further strengthened by selfitis activity, which not only represents an apparent gratifying means in being connected with others by posting selfies on social media but may also promote the addictive use of social media [33,75].

## 5. Conclusions

The inclusion of this selfitis behavior as an underlying mechanism in the significant relationships between dark traits and social media addiction corroborated (i) previous findings on these associations [13,18,19] irrespective of the type of social media platforms [22]; (ii) the three theoretical models predicting an elevated social media use by narcissistic individuals [28]; and (iii) the Uses and Gratifications theory [12], therefore adding empirical support to the research on selfitis behavior considered as a bridge which links the existing needs of self-concept by using a gratifying medium. Despite of some limitations, such as the relatively low reliability for each of the measures of the dark triad traits, the sample size, and the convenience sampling method that do not allow data generalization, self-report questionnaires that are subject to social desirability bias, and the cross-sectional nature of the data that could not determine the causality of the relationships, the present study testifies that the Italian version of the selfitis behavior scale can be considered a psychometrically robust instrument, whose construct shed much light into the mechanisms and dynamics underlying the onset of social media addiction. Further studies should replicate the research taking into account methodologically the need not only to find out the independent contribution of each trait to the outcomes, but also to control gender differences in the relationship between dark traits and online behavioral addictions. 

## Figures and Tables

**Table 1:** Parallel and confirmatory factor analysis of the SBS: Factor loading and error variance.

	1	2	3	4	5	Error Variance
SBS3	0.672 (0.666)					0.763
SBS2	0.653 (0.618)					0.525
SBS1	0.644 (0.794)					0.669
SBS4	0.427 (0.697)					0.387
SBS11		0.661 (0.877)				0.417
SBS10		0.618 (0.873)				0.362
SBS5		0.363 (0.716)				0.846
SBS9		0.350 (0.906)				0.486
SBS7		0.343 (0.775)				0.513
SBS18			0.675 (0.662)			0.343
SBS8			0.457 (0.836)			0.250
SBS12			0.452 (0.759)			0.285
SBS6			0.447 (0.691)			0.490
SBS20			0.288 (0.596)			0.783
SBS15				0.697 (0.797)		0.623
SBS14				0.516 (0.674)		0.544
SBS16				0.375 (0.783)		0.330
SBS17				0.292 (0.770)		0.559
SBS19					0.656 (0.882)	0.835
SBS13					0.600 (0.760)	0.693
Cronbach alpha (α)	0.77	0.87	0.85	0.82	0.55	
McDonald’s omega (ω)	0.77	0.87	0.86	0.82	0.55	
AVE	0.45	0.57	0.54	0.53	0.39	
CR	0.78	0.87	0.85	0.82	0.54	
√AVE						
F1	**0.673**					
F2	0.445	**0.754**				
F3	0.352	0.617	**0.735**			
F4	0.367	0.617	0.602	**0.725**		
F5	0.142	0.198	0.132	0.185	**0.662**	
FD	0.916	0.964	0.959	0.953	0.820	

F1 = social and emotional subjective wellbeing; F2 = self-confidence; F3 = self-presentation; F4 = need for reassurance/self-approval; F5 = autobiographical memories/documentation. CFA = confirmatory factor analysis; AVE = average variance extracted; values in brackets are factor loading from CFA. Values in diagonal (bold character) are the squared roots of the AVE.

**Table 2:** Test of the invariance of the Selfitis Behavior Scale across gender groups.

	χ^2^ (df)	CFI	TLI	RMSEA (90%, IC)	SRMR	Δχ^2^ (Δdf)	ΔCFI	ΔRMSEA	ΔSRMR
Male	207.34 *** (160)	0.967	0.961	0.046 (0.02, 0.06)	0.046	-	-	-	-
Female	327.93 *** (160)	0.920	0.905	0.081 (0.07, 0.09)	0.056	-	-	-	-
Configural	535.13 *** (320)	0.942	0.931	0.054 (0.05, 0.06)	0.049	-	-	-	-
Metric	555.52 *** (335)	0.940	0.932	0.053 (0.05, 0.06)	0.057	18.99 ^ns^ (15)	0.002	−0.001 (0.00, 0.00)	0.008
Scalar	602.95 *** (350)	0.932	0.926	0.056 (0.06, 0.06)	0.060	74.64 *** (15)	−0.008	0.003 (0.01, 0.00)	0.003

*** *p* < 0.001; ns = non-significant.

**Table 3:** Bootstrapped correlation matrix with 95% BCa confidence interval between the five dimensions of the SBS, BSMAS, number of selfies posts and number of friends on social media (Facebook, Twitter and Instagram).

	*r*	BCa 95% CI	R^2^
F1	0.777 **	0.724–0.824	0.604
F2	0.897 **	0.879–0.915	0.805
F3	0.842 **	0.811–0.869	0.709
F4	0.869 **	0.832–0.882	0.755
F5	0.668 **	0.642–0.731	0.446
BSMAS	0.507 **	0.429–0.580	0.257
Selfie posts	0.123 **	0.030–0.221	0.015
Friends on SM	0.150 **	0.061–0.235	0.023

F1 = social and emotional subjective wellbeing; F2 = self-confidence; F3 = self-presentation; F4 = need for reassurance/self-approval; F5 = autobiographical memories/documentation; BSMAS = Bergen Social Media Addiction Scale; ** *p* < 0.001; *r* = Pearson’s coefficient, R^2^ = coefficient of determination.

**Table 4:** Bootstrapped correlation matrix with 95% BCa confidence interval between the five dimensions of the SBS, BSMAS, number of selfies posts and number of friends on social media (Facebook, Twitter and Instagram).

	F1	BCa 95% CI	F2	BCa 95% CI	F3	BCa 95% CI	F4	BCa 95% CI	F5	BCa 95% CI
BSMAS	0.411 **	0.327–0.499	0.470 **	0.385–0.549	0.422 **	0.323–0.506	0.468 **	0.382–0.547	0.306 **	0.221–0.384
Selfie posts	0.269 **	0.190–0.348	0.256 **	0.165–0.338	0.181 **	0.091–0.267	0.200 **	0.144–0.280	0.148 **	0.056–0.236
Friends on SM	0.189 **	0.106–0.266	0.150 **	0.062–0.229	0.109 *	0.031–0.185	0.076	−0.007–0.160	0.130 **	0.044–0.210

F1 = social and emotional subjective wellbeing; F2 = self-confidence; F3 = self-presentation; F4 = need for reassurance/self-approval; F5 = autobiographical memories/documentation; BSMAS = Bergen Social Media Addiction Scale; * *p* < 0.05; ** *p* < 0.001.

**Table 5:** Zero-order and partial correlations among the variables of interest for the total sample and for each gender group.

	Total Sample (*n* = 490)		Males (*n* = 228)		Females (*n* = 262)	
	SBS	BSMAS	SBS	BSMAS	SBS	BSMAS
Narcissism	0.198 ***	0.146 **	0.191 **	0.097	0.270 ***	0.224 ***
	0.119 **	0.073	0.147 *	0.046	0.145 *	0.138 *
Machiavellianism	0.113 *	0.151 **	0.063	0.129	0.218 ***	0.224 ***
	−0.018	0.069	−0.062	0.076	0.073	0.097
Psychopathy	0.248 ***	0.179 ***	0.287 ***	0.150 *	0.202 ***	0.267 ***
	0.174 ***	0.099 *	0.245 ***	0.101	0.148 *	0.129 *

* *p* < 0.05; ** *p* < 0.01; *** *p* < 0.001. Zero-order correlations (r) are shown in the first row; partial correlations (sr) of each dark trait controlled for the other two dark traits are shown in the second row.

## Appendix A

**Selfitis Behavior Scale**
1. Taking selfies gives me a good feeling to better enjoy my environment
Fare i selfie mi fa provare una bella sensazione per divertirmi di più nel mio ambiente/contesto.
2. Sharing my selfies creates healthy competition with my friends and colleagues
La condivisione dei miei selfie genera una sana competizione con i miei amici e colleghi
3. I gain enormous attention by sharing my selfies on social media
Attiro moltissima attenzione con la condivisione dei miei selfie sui social media
4. I am able to reduce my stress level by taking selfies
Riesco a ridurre il mio livello di stress facendo selfie
5. I feel confident when I take a selfie
Mi sento sicuro di me stesso quando faccio un selfie
6. I gain more acceptance among my peer group when I take selfie and share it on social media
Sono accettato di più nel mio gruppo di pari quando faccio un selfie e lo condivido sui social media
7. I am able to express myself more in my environment through selfies
Con i selfie riesco a esprimere di più me stesso nel mio ambiente/contesto
8. Taking different selfie poses helps increase my social status
Fare pose differenti nel selfie aiuta a migliorare il mio stato sociale
9. I feel more popular when I post my selfies on social media
Mi sento più popolare quando posto i miei selfie sui social media
10. Taking more selfies improves my mood and makes me feel happy
Fare più selfie migliora il mio stato d’animo e mi fa sentire felice
11. I become more positive about myself when I take selfies
Divento più positivo riguardo a me stesso quando faccio selfie
12. I become a strong member of my peer group through selfie postings
La mia appartenenza al gruppo dei pari si rafforza postando i selfie
13. Taking selfies provides better memories about the occasion and the experience
Fare selfie fa ricordare meglio gli eventi e le esperienze
14. I post frequent selfies to get more ‘likes’ and comments on social media
Posto frequentemente selfie per avere più “mi piace” (likes) e commenti sui social media
15. By posting selfies, I expect my friends to appraise me
Postando selfie mi aspetto apprezzamenti dagli amici
16. Taking selfies instantly modifies my mood
Fare selfie modifica all’istante il mio stato d’animo
I7. I take more selfies and look at them privately to increase my confidence
Faccio più selfie e li guardo privatamente per aumentare la fiducia in me stesso
I8. When I do not take selfies, I feel detached from my peer group
Quando non faccio selfie mi sento distaccato dal mio gruppo dei pari
19. I take selfies as trophies for future memories
Faccio selfie come trofei per ricordi futuri
20. I use photo editing tools to enhance my selfie to look better than others
Utilizzo strumenti di fotoritocco per migliorare il mio selfie e apparire meglio degli altri
